# Effect of Fiber Laser Irradiation on the Shear Bond Strength between Acrylic Resin and Titanium

**DOI:** 10.1155/2019/5452919

**Published:** 2019-09-03

**Authors:** Fatih Mehmet Korkmaz, Selin Aycan

**Affiliations:** Department of Prosthodontics, Faculty of Dentistry, Karadeniz Technical University, 61080 Trabzon, Turkey

## Abstract

**Objectives:**

The aim of this study is to investigate the shear bond strength of an acrylic resin to titanium after different surface treatment methods.

**Material and Methods:**

A total of seventy-two disc-shaped specimens (10 mm × 10 mm × 2 mm) were prepared from titanium alloy. The specimens were randomly allocated to six equal groups: Group S (sandblasting), Group MP (metal primer), Group 10W (fiber laser 10 W), Group 20W (fiber laser 20 W), Group 10WMP (fiber laser 10 W+metal primer), and Group 20WMP (fiber laser 20 W+metal primer). All of the specimens were thermocycled up to 5000 cycles. After thermal cycling, a shear bond strength test was conducted. The shear bond strength data were analyzed with one-way ANOVA and Tukey's post hoc pairwise comparisons (*p* < 0.05).

**Results:**

While the highest values were determined in Group MP, the lowest values were observed in Group S. Additionally, Group MP exhibited significantly higher shear bond strength values than any of the other groups (*p* < 0.05) except Group 10WMP. Similar results were observed between Group MP and Group 10WMP (*p* > 0.05). The groups in which a metal primer was applied (Group MP, 10WMP, and 20WMP) showed significantly higher values than Group S. The shear bond strength values of Group 10W and Group 20W were similar.

**Conclusions:**

The application of a metal primer significantly improved the bond strength of acrylic resin to titanium. Fiber laser application may be an alternative method to sandblasting for improving the bond strength of acrylic resin to titanium.

## 1. Introduction

The allergic and toxic potentials of the alloys used in dentistry have necessitated the search for alternative metals. Titanium is a material that has low density, high resistance, especially resistance to corrosion, thermal transmittance, and perfect biocompatibility [1–5]. Due to the advantages brought about by these properties, titanium is now used frequently in prosthetic applications in dentistry [6]. Pure titanium and titanium alloys are used as a framework material in dentistry, including in crown-bridge restorations, implant-supported dentures, and removable dentures [3, 6, 7]. Titanium's high casting temperature and high level of chemical activity cause problems in casting [7]. Due to developing technologies, titanium is now produced with computer-aided design/computer-aided manufacturing (CAD/CAM) systems, and problems that occurred during casting have been eliminated [7]. Removable partial dentures are generally produced with the combined use of polymethyl methacrylate (PMMA) and metal [6, 8]. At the end of the follow-up process of a clinical trial that involved removable partial dentures with a titanium base, it was found that there were no clinical problems and that the only technical problem encountered was inadequate adhesiveness with acrylic resin [1]. The success of the bond between metal alloys and PMMA allows the denture to be long lasting [3]. The complication that is frequently encountered with these materials is the debonding of the acrylic resin from the metal [3, 6, 8]. The reason for this debonding is the difference between the thermal expansion coefficients of the metal alloy and the acrylic resin and the shrinking of the acrylic resin due to polymerization [3, 6, 8, 9]. The insufficient bond between these two materials causes microscopic gaps that can lead to serious clinical problems [9, 10]. These gaps can lead to microleakages, staining, accumulation of microorganisms in the area, breakages in the acrylic resin, and in time, unfavorable soft tissue response due to microorganisms [3, 6, 8–10]. Therefore, various mechanical and chemical methods have been employed to enhance the adhesive bonding of acrylic resins to metal alloys. Electrolytic etching, chemical etching, silica coating, spark erosion, laser application, sandblasting with Al_2_O_3_, and the application of metal primers are the most commonly used methods developed for this purpose [2, 3, 6–12].

The technique of sandblasting with alumina particles is widely used for enhancing the bond between the metal alloys and PMMA [6, 13]. It has been reported in various studies that having been a chemical method, the application of various adhesive primers is a successful method that eliminates the need for mechanical surface treatment and that enhances the bond between the PMMA and the metal [2, 3, 5, 9, 10]. The metal primer application containing 10-methacryloyloxydecryl dihydrogen phosphate (MDP) is one of the adhesive primer applications that creates an excellent chemical bond between the PMMA and the metal [2, 3, 8, 11]. In some previous studies, it was reported that the use of MDP-containing primers was more effective in enhancing the bond between the metal and the PMMA than other primers [3, 4, 11, 14].

Due to the development of dental technology, lasers are commonly used in dental procedures and in the surface treatment of dental materials. Studies have shown that laser applications, such as CO_2_ (carbon dioxide), Er:YAG (erbium, yttrium, aluminum, garnet), Nd:YAG (neodymium-doped, yttrium, aluminum, garnet), and Er,Cr:YSGG (erbium, chromium: yttrium-scandium-gallium-garnet) alter the material surface and create a larger area for bonding [9, 15–20]. In their study, Murray et al. [16] demonstrated that laser etching the surface of a NiCr alloy produced better results than sandblasting with regard to resin bonding. One study reported that the application of an Nd:YAG laser on a metal surface increased the strength of a bond between Co-Cr and polymethyl methacrylate [9]. However, recent studies have shown that higher output power applications caused cracks and defects on the surface of the material and caused an undesirable increase in temperature [19, 21].

Fiber lasers, which are the most recent products of the current laser technology, have an area of application in dentistry as well because they are compatible with industrial and biomedical applications and they can produce high power in a very short period of time [21]. Fiber lasers exhibit some important advantages over previous technologies with regard to source brilliance, oscillating mode stability, efficiency, possibility of monolithic packaging, and low maintenance costs [22, 23]. Fiber lasers quickly and powerfully affect surfaces while causing less mechanical and thermal damage compared to other lasers [22–25]. Ultrafast fiber lasers are mainly used in dentistry to modify the surface of implants [22, 26, 27]. Since laser parameters have the capability to influence and alter the surface microstructure, lasers may be an alternative method for surface treatment for the purpose of enhancing the bond between various materials [23]. One study showed that a femtosecond laser application increased the bond between both ceramic and metal brackets and zirconium oxide [25]. Ates et al. [18] reported that ultrafast fiber laser applications increased the bond strength between resin cement and titanium and that ultrafast fiber lasers may be an alternative method for roughening metal surfaces.

An examination of the literature reveals that, although many researchers have examined various studies on enhancing the bond between titanium and PMMA, there have been no studies on the use of fiber lasers in this area. The purpose of this study is to examine the influence of fiber laser, sandblasting, and metal primer applications on the bond strength between titanium and PMMA. The hypothesis of the study is that fiber laser applications will not lead to better results than the other methods.

## 2. Materials and Methods

The materials used in the study are given in Table 1. Seventy-two disc-shaped titanium alloy (Ti6Al4V) (Kobe Steel Co., Japan) samples (15 mm diameter and 2 mm height) were obtained by the use of CAD/CAM machine-milled technology. The surface irregularities of the samples were corrected in running water by use of a 600-grit silicon carbide paper (CarbiMet, Buehler, Illinois, USA). All samples were then washed for 5 minutes in an ultrasonic bath and dried for 30 seconds. The samples were divided into 6 groups as follows according to the type of surface treatment method (*n* = 12):

- Group S: sandblasting with 50 *μ*m Al_2_O_3_
- Group MP: metal primer application
- Group 10W: nanosecond fiber laser application (10 W)
- Group 20W: nanosecond fiber laser application (20 W)
- Group 10WMP: nanosecond fiber laser (10 W)+metal primer application
- Group 20WMP: nanosecond fiber laser (20 W)+metal primer application

The sandblasting process was performed for 15 seconds at 0.5 MPa air pressure from a distance of 10 mm by applying 50 *μ*m Al_2_O_3_ particles.

**Figure 1:**
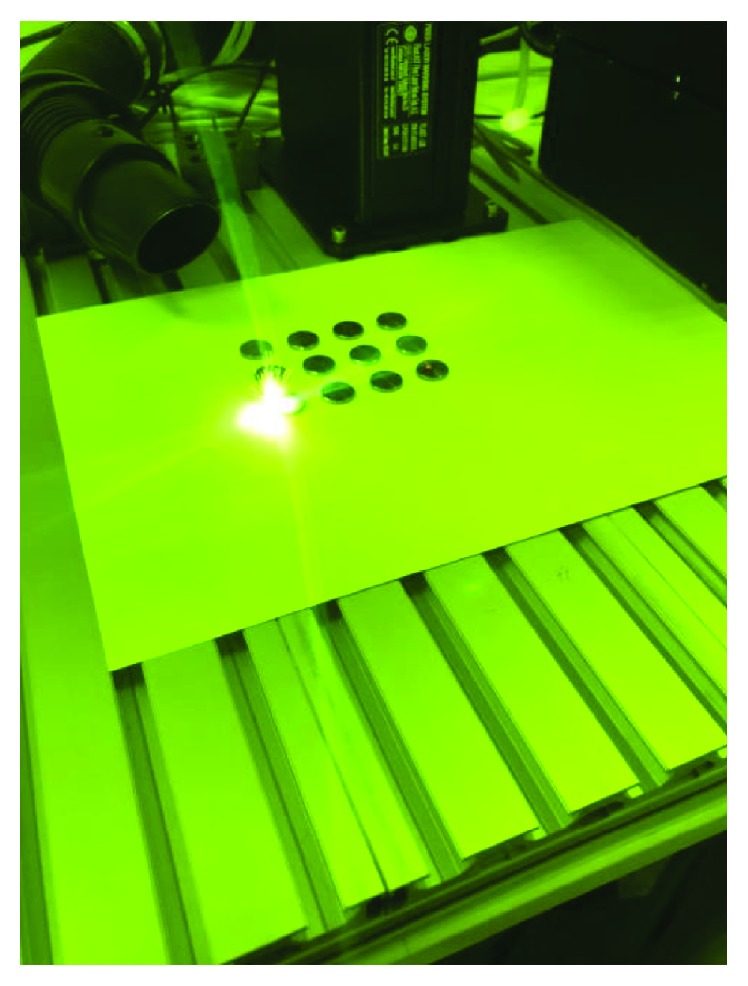
Fiber laser application (FiberLAST Inc., Ankara, Turkey).

For the nanosecond fiber laser application, a 1064 nm wavelength ytterbium (Yb) fiber laser (FiberLAST Inc., Ankara, Turkey) system device was used (Figure 1). For the surface preparation process of the titanium samples with laser, the pulse energy was 1 mJ, the repetition rate was 100 kHz, the pulse length was 100 ns (ultrashort pulse), and the maximum average output power was 10 W or 20 W depending on the test group. A 7 mm beam is ordinarily produced from the system collimator outlet. The laser beam with a 7 mm output diameter was focused on a 20 *μ*m spot area, and the samples were placed on a surface that could be moved in two dimensions with a computer. The lens used in this system is able to determine the focal point independent of the operator and adjust the distance manually or automatically. The collimator with back reflection protection that was attached to the outer laser protected the material from back reflection. The air inside the fiber laser system was used for the cooling process during the procedure.

After surface preparation, a typical sample surface from each group of surface treatments was visually examined under a scanning electron microscope (SEM; Jeol, JSM 6610, Tokyo, Japan). SEM images of the samples were obtained at various magnifications to investigate the surface topography better.

Titanium samples were embedded into autopolymerizing acrylic resin (Imicryl, Konya, Turkey) by way of sample silicone molds, with one surface exposed following surface preparation. A total of seventy-two wax discs (8 mm × 2 mm) were prepared using a stainless steel mold. The disc-shaped wax specimens were located in the center of titanium specimens and attached by applying pressure with fingers. The specimens were packed in flasks with investment. After the wax was removed by using standard boil-out procedures, the flasks were cooled at room temperature. The samples for Group MP, Group 10WMP, and Group 20WMP were set aside for the metal primer to be applied to their surfaces. For the metal primer application, the metal primer (Z-Prime PLUS, ZPP; Bisco, Schaumburg, IL, USA) was applied for a duration of 15 seconds with a single-use brush, in one layer, so that the entire surface was coated. Then, the specimens were dried in oil-free air for 5 seconds, and a waiting period of 5 minutes was observed.

Heat polymerized acrylic resin (Meliodent Heat Cure, Heraeus Kulzer GmbH, Hanau, Germany) was applied on all samples in accordance with the manufacturer's recommendations. Once polymerization was complete, the samples in the flask were left to cool at room temperature. Once removed from the flask, the samples were kept for 24 hours in 37°C water, and the shear bond strength (SBS) values were examined after the samples were thermally cycled 5000 times between 5°C and 55°C, with a dwell time of 30 seconds in each bath. The shear test was carried out in the universal test device (Model 3340, Instron Corporation, England) at a speed of 0.5 mm/min, and the SBS values were calculated in megapascals (MPa) by dividing the failure load (*N*) by the bond surface area of the acrylic resin (mm^2^).

After the shear testing, the samples were examined under a scanning electron microscope (Jeol, JSM 6610, Tokyo, Japan) at 8x magnification to identify their failure types. The failure types were determined to be adhesive if there is failure between the titanium and resin interfaces, cohesive if there is failure within the acrylic resin itself, and mixed if some areas displayed cohesive failure and other areas displayed adhesive failure. For a more detailed examination, the chemical analyses of the surface structures of the samples were assessed with energy distribution X-ray spectroscopy (EDS).

Statistical analyses were carried out with a statistical software program (SPSS for Windows 17.0; Chicago, IL). The normality of the data was analyzed with the Shapiro-Wilk test. One-way ANOVA and Tukey's post hoc pairwise comparisons were used to analyze the data with a confidence interval of 95%.

## 3. Results

The mean values of SBS and the standard deviations of each surface treatment process and the results of each group's failure type are shown in Table 2. It was found in this study that Group MP had the highest bond strength value (23.14 ± 5.89 MPa) and Group S had the lowest value (7.00 ± 2.45 MPa). Group MP produced statistically better results than all groups except for Group 10WMP (*p* < 0.05). Although the results of Group MP were higher than those of Group 10WMP, the difference was statistically insignificant (*p* > 0.05). Both fiber laser groups (Group 10WMP and Group 20WMP) that had additional metal primer application produced better results than Group S (*p* < 0.001). Though the individual fiber laser-applied groups (Group 10W and Group 20W) produced better results than Group S, this difference was not statistically significant (*p* > 0.05). No significant differences in bond strength were determined between fiber laser groups, although Group 20W revealed slightly higher bond strength values (*p* > 0.05).

**Figure 2:**
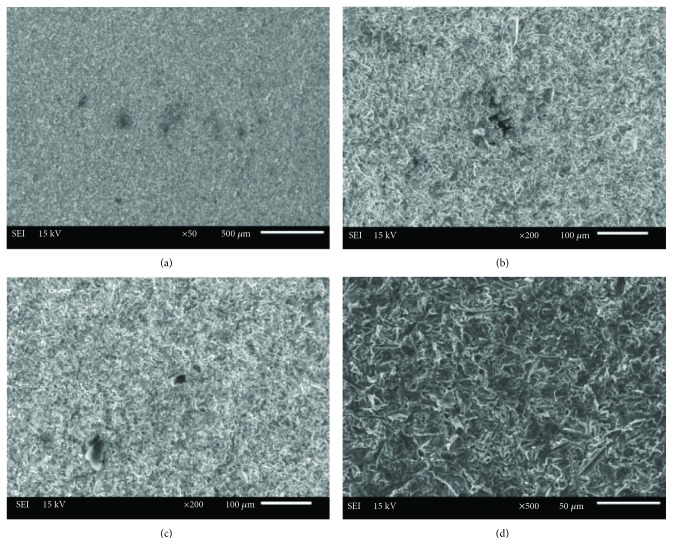
SEM images of titanium surface after sandblasting: (a) ×50 magnification, (b, c) ×200 magnification, and (d) ×500 magnification.

**Figure 3:**
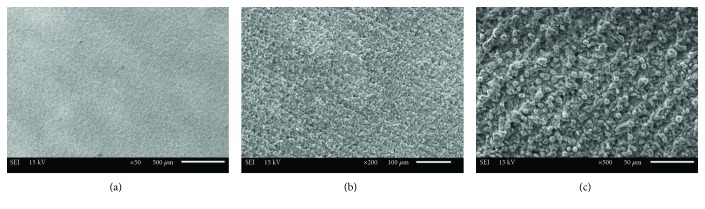
SEM images of titanium surface after nanosecond fiber laser application (10 W): (a) ×50 magnification, (b) ×200 magnification, and (c) ×500 magnification.

**Figure 4:**
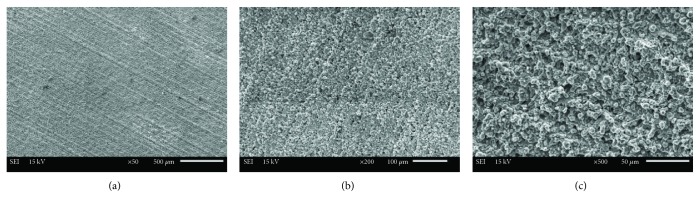
SEM images of titanium surface after nanosecond fiber laser application (20 W): (a) ×50 magnification, (b) ×200 magnification, and (c) ×500 magnification.

The SEM images of the titanium surface according to the surface treatment methods are shown in Figures 2–4. The sandblasted surfaces had an isotropic topography, which was irregular, with numerous cavities and clearly sharp-edged rims, undercuts, and protruding particles (Figures 2(a)–2(d)). The sandblasted surfaces demonstrated pore and groove formation. Some surface defects, such as pores and cracks, were found on the surface of sandblasted specimens (Figures 2(a)–2(c)). Different pore sizes and distribution were observed (Figures 2(b) and 2(c)). The SEM images of fiber laser-applied specimens revealed a homogenous rough surface with many holes and irregularities (Figures 3 and 4). This surface includes the formation of nanoripples/structures in the produced dimples and grooves (Figures 3 and 4). The presence of thermal damaging effects, such as melting, burning, and cracks, was not detected.

**Figure 5:**
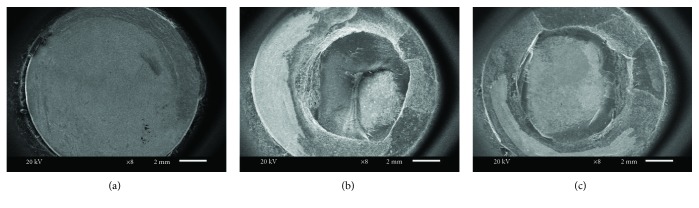
SEM images of titanium surfaces after shear bond testing (at ×8 magnification): (a) adhesive failure at metal/acrylic resin interface in a sandblasted specimen, (b) cohesive failure at the acrylic resin layer in a metal primer applied specimen, and (c) mixed failure in a fiber laser (20 W)+metal primer applied specimen.

The SEM images of the bond failures of some groups are shown in Figure 5. When the fractured samples were examined under a SEM after the shear bond test, it was found that there was a high rate of adhesive failure in all the groups (except for Group MP). Group MP had 58% mixed, 34% cohesive, and 8% adhesive failures. There were no cohesive failures in any group excluding Group MP. While the highest ratio adhesive failure mode was seen in Group S, the lowest was seen in Group MP. Both fiber laser groups (Group 10W and Group 20W) demonstrated similar failure modes.

**Figure 6:**
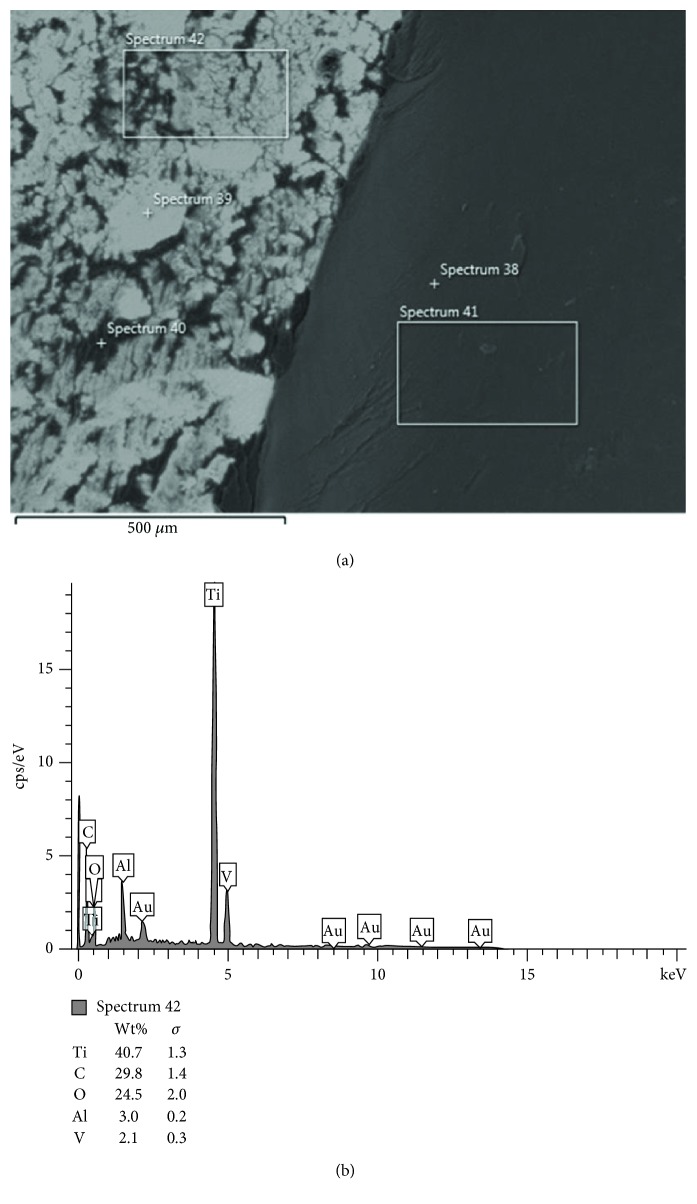
SEM images and corresponding EDS spectra of titanium surface in a metal primer applied specimen. (a) Cohesive failure at the acrylic resin layer (at ×300 magnification). (b) Number 42 EDS spectra reveals carbon (c) and gold (Au) elements related to acrylic resin on the titanium surface.

When the elementary structure of the surface of the metal primer-applied sample was examined with EDS after the shear test, it was observed that acrylic resin remained on the surface of the titanium (Figure 6). This determination was based upon the observation of high levels of carbon and low levels of Au element. Au element that is observed here was included in PMMA to increase thermal conductivity, to improve optical properties, and to increase transverse strength [28]. This finding also supported the fact that the failure of the metal primer group was a cohesive failure.

## 4. Discussion

This study investigated the effect of nanosecond fiber laser application at different power outputs, sandblasting with aluminum oxide, and the metal primer application on the bond strength between PMMA and titanium alloy. In this study, it was shown that the application of the metal primer produced the best results, the application of a fiber laser produces the same results as sandblasting, and the application of a fiber laser combined with a metal primer produced statistically better results than those produced by sandblasting. Therefore, our hypothesis has been partially rejected.

The successful bond between titanium and the acrylic resin in removable dentures with a titanium metal framework is the most important factor that determines long-term clinical success of removable partial dentures [3, 6]. Examination of the literature reveals that there are various studies regarding the enhancement of the bond between the titanium and the acrylic resin [1–4, 6, 8, 9]. The process of sandblasting with alumina increases surface roughness, bonding surface area, and surface energy. It also forms a chemical bond between the acrylic resin and metal alloys [8]. In a previous study, it was proved that the size of the alumina particles affects bond strength and that pretreatment with alumina particles that are larger results in a stronger bond than pretreatment with smaller particles [6]. In the mentioned study that included sandblasting with 50 *μ*m alumina particles, the bond failures were all adhesive, and the failures that occurred when sandblasting was carried out with 250 *μ*m were predominantly cohesive [6]. Similar findings were reported in some previous studies [12, 29, 30]. In the present study, it was reported that the results of sandblasting with 50 *μ*m alumina particles were the lowest among all the groups. The size of the PMMA particles that are the main component of polymeric resin is approximately 100 *μ*m, and the particles of this size are not able to fully penetrate a surface that has been roughened with small Al_2_O_3_ particles [12]. In addition, alumina particles that adhere to the metal surface inhibit the flowing of PMMA particles freely over the rough surface and hinder a strong bond [9, 12]. The aforementioned factors may explain the low bond strength values obtained in our study. A previous study emphasized that the sandblasting process is insufficient for a strong bond and that bonding methods involving chemical adhesion are also required [8].

Currently, the application of an adhesive primer to increase the strength of the bond between adhesive resins and metals has been investigated in various studies [3–5, 9, 14, 31]. Yoshida et al. [14] reported in their study that the functional monomers of adhesive primers interact with an oxide layer created on the metal surface and this interaction increases the bond strength of metal to acrylic resin. In a previous study, which examined the SBS and leakage of PMMA to dental alloys, it was reported that adhesive application improved bond strength and decreased the distance of dye penetration in the metal acrylic interface [31]. It was previously reported in some studies that the application of metal primers containing the phosphate group (including MDP monomers) on the metal surface enhances the bond between the acrylic resin and the metal [3–5, 7, 8, 32]. In their study in which they examined 5 different metal primers, Ohkubo et al. [4] showed that the application of a metal primer enhanced the bond between acrylic resin and titanium or Co-Cr alloy and that the metal primer containing MDP was the most effective primer. The oxide layer that forms on the surface of basic metal alloys chemically bonds with the dihydrogen phosphate component of the MDP monomer, and this mechanism provides a strong chemical bond between the resin and the metal alloys [5, 7–9, 33]. In some previous studies, applying a metal primer after sandblasting with Al_2_O_3_ particles resulted in higher bond strength values between the metal and the acrylic resin compared to samples on which the metal primer was not applied [11, 32]. The findings of our study support the findings of other studies in that the bond strength values of the group to which a metal primer was applied were the highest among all the other groups (23.14 ± 5.89). The increase in bond strength is caused by the chemical bonding of the dihydrogen phosphate group in the MDP monomer within the metal primer and the titanium oxide on the surface of the titanium by way of hydrogen bridges [5, 34]. This bond was most likely actualized by the covalent or ionic concentration of dihydrogen oxide [5, 34]. The bond failure types also support the results of the bond strength test. Cohesive failures were determined at a rate of 34% in the metal primer group that had the highest bond strength, and none of the other groups displayed cohesive failures. The rate of adhesive failures (8%) was the lowest in this group. The sandblasting group had the lowest bond strength, and the assessment of failure types showed that adhesive failures had the highest rate (84%). In the groups other than the sandblasting group and the metal primer group, the failure types that were determined were predominantly adhesive and mixed.

Various dental lasers, such as Er:YAG; Nd:YAG; CO_2_; and Er,Cr:YSGG, have been used to roughen the metal surfaces in dentistry [23]. Some studies have shown that laser applications are effective in increasing bond strength, and some have not found any differences in results compared to other surface treatments. Venkat et al. [35] showed in their study that the application of an Nd:YAG laser on a titanium abutment surface enhanced the bond between the temporary acrylic crown and the abutment. In a study conducted by Akın et al. [17], it was indicated that the Nd:YAG laser application may be an alternative method to sandblasting with Al_2_O_3_ for enhancing the bond strength between titanium and porcelain. Yılmaz et al. [9] determined that the application of an Nd:YAG laser on a metal alloy surface enhanced its bond to PMMA and indicated that applying a metal primer after the laser application further enhanced the bond strength. A previous study reported that the Nd:YAG laser application was better than the application of acid etching, and the laser enhanced the bond between titanium and low temperature porcelain; however, the laser was not a better method than sandblasting [36]. Because in some studies the surface treatment with dental lasers did not achieve the desired effect on the material, more powerful lasers were required to increase the effect. Fiber lasers have a good beam quality, are absorbed with ease into metal surfaces, and provide surface treatment without thermal effects due to their short pulse length [25, 27, 37, 38]. This laser has extremely high repetition rates, which makes ablating cooling possible, reduces the laser pulse energies for needed ablation, and increases the efficiency of the removal process [25, 27, 37]. These advantages have led to fiber lasers being preferred in material processing in recent years [23, 24, 37, 38]. However, studies on their use in dentistry are limited. Kara et al. [38] showed that femtosecond lasers resulted significantly higher bond strength of resin cement to zirconia than the other lasers. While one study demonstrated that a femtosecond laser treatment enhanced the bond between ceramic and metal brackets with zirconium [25], another study showed that a femtosecond laser application did not enhance the bond between a fiber post and resin cement [24].

In the literature, no previously published studies take into account the use of a nanosecond fiber laser as a surface treatment method to enhance the bond between titanium and acrylic resin. In the present study, while the application of a fiber laser alone did not produce better results than sandblasting, significant increases in bond strength values were detected when a metal primer was applied after the fiber laser application. These data are in agreement with a study by Yılmaz et al. [9], which reported that when a metal primer was applied together with a laser application, better results were achieved than when only a laser was applied. However, unlike our findings, those authors found that the use of a metal primer together with laser application produced better results than the application of a metal primer alone. The reason for this disparity could be that the researchers sandblasted the samples before the laser application in their study. Additionally, this discrepancy could be attributed to a difference in the type of laser: we used a nanosecond fiber laser, whereas the cited study used an Nd:YAG laser. In their study, Saygın et al. [39] revealed that an Nd:YAG laser produced better results, an Er:YAG laser produced similar results, and a Ho:YAG laser produced lower results compared to sandblasting. As it can be clearly inferred, the type of laser affects the bond strength. In our study, the use of a fiber laser with a strong effect may have resulted in stress accumulating in the surface due to mechanical effects. The stress accumulating on the metal surface may increase surface tension and prevent the full wettability of the adhesive on the surface and, therefore, result in the increase in bond strength being lowered.

Although our study found that laser application alone produced better results than the sandblasting application, this difference was not statistically significant (*p* > 0.05). In a study conducted by Madani et al. [40], it was found that the application of an Nd:YAG laser with 6 W or 8 W output power on the surface of the metal did not increase the bond of the resin cement to the metal and that the sandblasting application with Al_2_O_3_ produced better results than the laser application. The decrease in the bond strength after the laser treatment has been explained by a change in the surface characteristics of the metal due to the laser application [40]. Additionally, in a study conducted by Kunt et al. [41], it was found that the application of an Er:YAG laser did not increase the roughness of the metal surface in comparison to the group that had no surface treatment. In contrast to this study, it was demonstrated that the use of an Nd:YAG laser produced better results than sandblasting with Al_2_O_3_ regarding the bond between the composite resin and the NiCr metal alloy surface [42]. According to Grover and Nandlal [43], the fiber laser application could be regarded as an alternative to sandblasting. In the findings of a previous study in which we investigated the influence of the ultrafast fiber laser irradiation to the bond strength between the resin cement and the titanium, we determined that sandblasting with Al_2_O_3_ and the fiber laser application produced similar results in regard to bond strength, which is supportive of the results of the current study [18]. In a study conducted by Erdur and Basciftci [44], it was found that the application of a Ti-sapphire laser produced higher values than sandblasting, acid etching, and other laser applications regarding the bonding to orthodontic brackets to ceramic surfaces. The applications of fiber lasers with various outputs and energies for various durations are promising regarding the production of better results than other surface treatments. There is a great need for further studies on this subject.

This study has found that the nanosecond fiber laser application and sandblasting with Al_2_O_3_ produced similar results. The application of a metal primer after the laser application produced better results than both laser groups. In addition, the application of a metal primer alone produced the best results among all the groups. Therefore, it is possible to say that if we compare micro mechanic and chemical methods, the chemical agents are more effective. Similarly, in a study conducted by Maruo et al. [45], the processes of plasma beam application and sandblasting with Al_2_O_3_ produce weaker results than the metal primer application with regard to the bond between the acrylic resin and the cobalt-chrome alloy. Several studies demonstrated that the only mechanical methods used in some studies were insufficient and that the application of chemical agents, such as silane and metal primers, was effective in enhancing the bond, which is supportive of our study [2, 3, 8, 9, 32, 33].

In previous studies, SEM examination revealed that the Nd:YAG and Er:YAG lasers produced undesirable effects on the surface of the metal alloys, such as narrow micro cracks, deep craters, pits, and melting of the material, as the result of laser beam strikes [41, 42]. On the other hand, when compared to other lasers, a nanosecond laser has the fewest undesirable effects on the surface. One study reported that a fiber laser presents a dimpled geometry with a regular shape that is almost the optimal shape for friction reduction applications [46]. In the present study, in the nanosecond fiber laser specimens, there was no heat-affected zone that could be detected by SEM. Reducing the heat-affected zone is important, since these heat-affected zones are more vulnerable to the formation of cracks that reduces the material properties [22]. Although some surface defects, such as pores and micro cracks, have occurred on the surface of the titanium specimens during sandblasting, nonremovable alumina particles that are embedded into the surface could be responsible for the chemical bonding of acrylic resin [41].

One limitation of the present study is that only one type of laser (nanosecond fiber laser) and metal primer were tested. Since various factors affect the results, future studies using different types of lasers with different output parameters and different metal primers may lead to better results. Another limitation of this study was that other factors, such as pH changes and dynamic fatigue loading that could significantly influence the results, were not evaluated in this study, although thermocycling was performed. The influence of these factors must be investigated in further studies. The aforementioned benefits of fiber lasers, as well as their success in clinical applications, have not been entirely demonstrated due to their high cost and physical size. A great many long-term clinical studies are needed for fiber lasers to become a routine clinical procedure.

## 5. Conclusions

Within the limitations of the present study, the following conclusions can be drawn:

1. Among the surface treatment methods tested, the metal primer application alone demonstrated the highest SBS values, whereas sandblasting demonstrated the lowest SBS values
2. The metal primer application combined with the nanosecond fiber laser improved the bond strength between acrylic resin and titanium
3. The nanosecond fiber laser application alone produced similar results to sandblasting. Therefore, the fiber laser could be an alternative to sandblasting for enhancing the SBS value between heat-polymerized acrylic resin and titanium

## Figures and Tables

**Table 1:** Materials used in this study.

Material type	Material name	Chemical composition	Manufacturer	Lot no.
Titanium	Kobelco	Ti6Al4V, grade 5, Ti 89.0%, Al 6.0%, V 4.0%	Kobe Steel Co., Japan	—
Aluminum oxide	Korox	99.6% aluminum oxide	Bego, Bremen, Germany	05450312
Metal primer	Z-Prime PLUS	BPDMA, HEMA, MDP, ethanol	ZPP; Bisco, Schaumburg, IL, USA	1700006318
Acrylic resin	Meliodent Heat Cure	Polymethyl methacrylate	Heraeus Kulzer GmbH, Hanau, Germany	R010034

BPDMA: biphenyl dimethacrylate; HEMA: hydroxyethylmethacrylate; MDP: 10-methacryloyoloxydecyl dihydrogen phosphate.

**Table 2:** Shear bond strength values (mean ± SD), statistical results, and failure types (%).

Groups	Shear bond strength (MPa)	Failure types	
Sandblasting	7.00 ± 2.45^A^	Adhesive	84%
		Cohesive	—
		Mixed	16%

Metal primer	23.14 ± 5.89^B^	Adhesive	8%
		Cohesive	34%
		Mixed	58%

Fiber laser (10 W)	9.70 ± 2.19^A^	Adhesive	67%
		Cohesive	—
		Mixed	33%

Fiber laser (20 W)	10.33 ± 2.50^A^	Adhesive	67%
		Cohesive	—
		Mixed	33%

Fiber laser (10 W)+metal primer	19.35 ± 4.51^B,C^	Adhesive	58%
		Cohesive	—
		Mixed	42%

Fiber laser (20 W)+metal primer	18.06 ± 4.85^C^	Adhesive	50%
		Cohesive	—
		Mixed	50%

Different superscript capital letters indicate significant differences (*p* < 0.05).

## Data Availability

The data used to support the findings of this study are available from the corresponding author upon request.
